# Teaching Nurse Practitioner Students About Polypharmacy Through a Lived Experience

**DOI:** 10.1093/geroni/igaa057.677

**Published:** 2020-12-16

**Authors:** Jennifer Kim, Kanah Lewallen, Taylor Boll

**Affiliations:** Vanderbilt University, NASHVILLE, Tennessee, United States

## Abstract

Polypharmacy (typically defined as the concomitant use of 5 or more medications) affects 40-50% of older adults in the U.S., and is associated with geriatric syndromes, a higher risk of medication non-adherence, and adverse drug events. Medication non-adherence is a common frustrating clinical issue for clinicians who provide care for older adult patients. Simultaneously, patients often find medication regimens to be complicated and confusing. This may contribute to medication non-adherence, which may further lead to adverse drug events and/or negative health outcomes. The more medications a patient is taking, the higher the risk for non-adherence. Thirty-eight students enrolled in an adult-gerontology primary care nurse practitioner program were given a bag of five mock medications that are commonly prescribed for older adults. Students were instructed to follow the directions on each of the bottles for approximately one month. A private messaging system was available for students if refills were needed or if they had questions about their medications. A debriefing session for this month-long, ungraded simulation was held, at which time students returned medication bottles. Pill counts were not analyzed, but all returned bottles contained mock medications. Approximately 52.6% of students estimated adhering to the medication regimen 0-24% of the time, whereas 26.3% reported an adherence rate of 25-50%. The most commonly cited barrier to adherence (55.3%) was “forgetfulness”. Nearly all students (89.5%) reported that the exercise “very much” increased their awareness of challenges patients face when managing medications, and 97% cited an increased awareness of ways to improve medication adherence.

